# Air Temperature and Gastroenteritis Among Rohingya Populations in Bangladesh Refugee Camps

**DOI:** 10.1001/jamanetworkopen.2025.5768

**Published:** 2025-04-18

**Authors:** Takuya Takata, Xerxes Seposo, Nasif Hossain, Kayo Ueda

**Affiliations:** 1 Department of Hygiene, Faculty of Medicine, Graduate School of Medicine, Hokkaido University, Sapporo, Japan; 2 School of Tropical Medicine and Global Health, Nagasaki University, Nagasaki, Japan; 3 Ateneo Center for Research and Innovation, Ateneo School of Medicine and Public Health, Ateneo de Manila University, Pasig, Philippines; 4 Nutrition and Clinical Services Division, International Centre for Diarrhoeal Diseases Research, Bangladesh, Dhaka, Bangladesh

## Abstract

**Importance:**

The Rohingya displaced population in Bangladesh is the largest stateless population in the world. Infectious diseases, such as gastroenteritis, respiratory infections, and fever, are among the major health problems the Rohingya population has faced. Although associations between gastroenteritis and air temperature have been reported in various regions, no study has yet been carried out among the displaced populations.

**Objectives:**

To evaluate the association between air temperature and risk of gastroenteritis among the forcibly displaced Rohingya population in refugee camps in Bangladesh.

**Design, Setting, and Participants:**

In this cross-sectional study, daily time series data derived from facility-based case reports were collected in 2 clinics organized by the UNHCR (United Nations High Commissioner for Refugees) in Kutupalong and Nayapara registered camps from January 1, 2019, to December 31, 2021. Statistical analysis was conducted from April 2023 to September 2024.

**Exposure:**

Hourly 2-m air temperature from ERA5-Land by the European Centre for Medium-Range Weather Forecasts.

**Main Outcomes and Measures:**

The daily number of gastroenteritis cases recorded in the camp clinics was the main outcome measure. Nonlinear lagged associations between daily temperature and gastroenteritis cases were modeled using a quasi-Poisson generalized linear model to account for overdispersion coupled with a distributed lag nonlinear model including a maximum 21-day lag. Covariates from the literature were adjusted in the model.

**Results:**

A total of 33 280 gastroenteritis cases (95% among individuals aged ≥5 years; 71% female) were recorded in Kutupalong and 31 165 gastroenteritis cases (99% among individuals aged ≥5 years; 67% female) were recorded in Nayapara. Further examination revealed a potential U-shaped curve in Kutupalong with minimum risk temperature (MRT) set at 26 °C. Cumulative relative risk (RR) at the 10th percentile temperature (21.1 °C) was 2.31 (95% CI, 1.18-4.65), while RR at 90th percentile temperature (28.5 °C) was 1.78 (95% CI, 1.24-2.56) relative to MRT. In Nayapara, a nearly linear risk increase was observed with decreasing temperature. Cumulative RR at the 10th percentile temperature (21 °C) was 1.32 (95% CI, 0.78-2.24), while the RR at the 90th percentile temperature (28.3 °C) was 0.75 (95% CI, 0.56-0.99). Lagged effects were delayed in nature. In Kutupalong, cold temperatures (10th percentile) were associated with statistically significant gastroenteritis risks at approximately 15 to 20 days (range: RR, 1.06 [95% CI, 1.00-1.13] to RR, 1.10 [95% CI, 1.00-1.21]). In Nayapara, gastroenteritis risks were correspondingly higher at longer lags (lag, 18 days; RR, 1.05 [95% CI, 1.00-1.10]).

**Conclusions and Relevance:**

In this cross-sectional study of the Rohingya displaced population in Bangladesh, cold temperatures were associated with an increase in the risk of gastroenteritis. It is important to understand the association of climatic factors with the health of displaced communities, whose population is expected to grow in the future.

## Key Points

**Question:**

How is air temperature associated with the risk of gastroenteritis among refugee populations?

**Findings:**

In this cross-sectional study that included 64 445 gastrointestinal cases among 2 Bangladesh refugee camps (33 280 gastroenteritis cases in Kutupalong and 31 165 gastroenteritis cases in Nayapara), the risk of gastroenteritis generally increased in colder temperatures.

**Meaning:**

As the refugee population is expected to grow in the coming years, it is crucial to understand the climate-related health risks they face to protect their well-being in the face of the escalating threats posed by a changing climate.

## Teaser

This cross-sectional study of the Rohingya displaced population evaluates the association between air temperature and risk of gastroenteritis in refugee camps in Bangladesh.

## Introduction

Forced displacement, driven by conflict, human rights violations, persecution, and disasters, continues to rise. A recent report by the United Nations High Commissioner for Refugees (UNHCR)^1^ revealed that more than 100 million people were forcibly displaced in 2022. Of these, 35.3 million people are refugees, with 29.4 million under UNHCR’s mandate and 5.9 million classified as Palestine refugees under the mandate of the United Nations Relief and Works Agency for Palestine Refugees in the Near East. Approximately 9.8 million reside in settlements managed by the UNHCR.^2^ In addition, projections indicate that approximately 1.2 billion individuals could face displacement by 2050 due to climate change–related events.^3^

Despite the looming threat, research on climate vulnerabilities among forcibly displaced persons (FDPs) remains scarce. Displaced populations tend to have higher crude death rates compared with nondisplaced populations, especially during the early phases of displacement and emergencies, due to factors such as poor living conditions, lack of access to health care, and increased risk of infectious diseases.^4,5^ Infectious diseases remain significant health threats to FDPs.

The Rohingya are among the largest stateless populations, comprising one-seventh of the global stateless populations.^6^ They are not considered to be citizens by the Myanmar government, and they migrated primarily to neighboring countries, notably Bangladesh, Malaysia, and Thailand (Forcibly Displaced Myanmar Nationals). Bangladesh has hosted several waves of Rohingya arrivals since 1948.^7^ In 2021, the escalation of unrest in Myanmar prompted a large number of Rohingya people to seek refuge in Bangladesh, bringing the total number of new arrivals to an estimated 918 898.^8^ In Cox’s Bazar, Bangladesh, there are 34 camps for the Rohingya population.^9^ These camps’ population density reaches 60 000 people per square kilometer, creating severe public health challenges.^10^

A recent study conducted at public health sector hospitals highlighted the prevalence of infectious diseases such as respiratory infections, abdominal ailments, viral fever, and diarrhea in Rohingya camps.^11^ Overcrowded and unsanitary living conditions, combined with the use of contaminated shallow wells and limited water sources during the dry season, exacerbate these health issues. A 2018 survey revealed that approximately 75% of household water sources were contaminated with fecal coliforms, with one-third containing *Escherichia coli*.^12^

Air temperature is significantly associated with diarrhea and enteric infections.^13,14^ A systematic review found that low air temperatures are associated with increased risk of viral gastroenteritis, including norovirus, rotavirus, and adenovirus,^14^ while in a different study, high air temperatures were associated with heightened risk of bacterial gastroenteritis caused by organisms such as *Salmonella* and *Campylobacter*.^15^ However, these findings were based on data from the general, nondisplaced population, which has little implication to FDP settings in refugee camps. Given the projected increase of FDPs and their health challenges, understanding the association of climate change with health is essential. Such insights will enable better preparedness for both FDPs and host community health systems in effectively responding to these climate-related health risks. Against this backdrop, this study aimed to evaluate the association between air temperature and gastroenteritis in FDP camps in Bangladesh.

## Methods

### Data Sources

#### Study Setting

This is a retrospective observational cross-sectional study of daily historical records of gastroenteritis and mean air temperature in 2 camp clinics in the Kutupalong and Nayapara camps from January 1, 2019, to December 31, 2021. Patients who visited the 2 clinics run by the UNHCR at Kutupalong and Nayapara, 2 government-run Rohingya camps sites in Ukhia and Teknaf Upazila, and Cox’s Bazar in Bangladesh (eFigure 1 in Supplement 1) were included in this study. During the midstudy period in 2020, the population size was 16 855 in Kutupalong and 22 589 in Nayapara.^16^ This study adhered to the Strengthening the Reporting of Observational Studies in Epidemiology (STROBE) reporting guideline for cross-sectional studies. Data used in this study were deidentified; thus the study was waived for ethics review per the Japanese Ethical Guidelines for Life Science and Medical Research Involving Human Subjects. Consent was not obtained because the data in this study come from a deidentified secondary dataset. Primary data collection was not done in this study.

#### Health Outcome Data

The health outcome was the daily number of gastroenteritis cases recorded in the camp clinics. Study data were registered using District Health Information Software 2 (DHIS2)^17^ obtained from the Ministry of Health and Family Welfare of the Bangladesh government^18^ with the registration system developed by the University of Oslo. Gastroenteritis was defined operationally as moderate to severe diarrhea, following World Health Organization (WHO) guidelines. Moderate diarrhea was characterized by a few to 10 loose or watery stools in a 24-hour period, while severe diarrhea involved more than 10 loose or watery stools within the same time frame. Diagnosis was based on the presence of symptoms, including moderate to severe diarrhea and, in some cases, additional manifestations, such as blood in the stool (bacillary dysentery), vomiting, abdominal pain, and fever. These criteria align with WHO standards for diagnosing and evaluating the severity of gastroenteritis.^19^

#### Environmental Data

Exposure data comprised the hourly 2-m air temperature from ERA5-Land by the European Centre for Medium-Range Weather Forecasts accessed through the Copernicus Climate Change Service Climate Data Store.^20^ ERA5-Land provides meteorological data at a 0.1-degree (approximately 9-km) gridded resolution. Total precipitation from ERA5-Land was adjusted as a potential confounder in the temperature-gastroenteritis association.^21^ Hourly time series from Coordinated Universal Time (UTC) ± 00:00 was converted to UTC + 06:00 to match the temporal timing of the exposure and was subsequently aggregated into daily mean values.

Temperature data generated from the ERA-5 Land reanalysis were validated with the National Oceanic and Atmospheric Administration monitoring data of the closest station (Shah Amanat International Airport, which is approximately 100 km north of the camp locations). A strong correlation (*r* = 0.955) was observed in the seasonality-adjusted model residuals (eFigure 2 in Supplement 1).

#### Covariates

Covariates, such as precipitation, long-term time trend, day of the week, holiday, population, COVID-19 pandemic, and reporting system indicator terms, sourced from the literature were adjusted in the model. Although the UNHCR has ensured access to basic health services during the COVID-19 pandemic,^22,23^ we decided to include a binary variable to address the potential association of the COVID-19 pandemic with case reporting, whereby the days before March 11, 2020 (when the pandemic was declared), were coded 0 and the days thereafter were coded 1.^24^ In addition, holidays were identified based on the Bangladesh government calendar, and for days of the week, a categorical variable was used, defining the country’s weekends as Friday and Saturday.

### Statistical Analysis

Statistical analysis was conducted from April 2023 to September 2024. The validation of exposure data was assessed through correlational analysis. Descriptive statistics for both exposure and health data from the camp clinics were summarized using mean and median values. Nonlinear lagged associations between daily temperature and gastroenteritis cases were modeled using a quasi-Poisson generalized linear model to account for overdispersion coupled with a distributed lag nonlinear model,^25,26,27^ which is parameterized as follows: log [*E*(*Y*)] = α + cb(Temp lag) + ns(Prcep, *df* = 3) + ns(Time, *df* = 7/y) + *N*_holiday_ + DOW + COVID19 + Reporting_indicator + offset [log (population)] + *Y*_(_*_t_* _− 1)_, where *E*(*Y*) is the expected number of daily reported cases of gastroenteritis; α is the intercept; cb(Temp∙lag) is the cross-basis function for the DLNM, with natural cubic splines of daily mean temperature and lags with a maximum lag of 21 days^28,29,30^ parameterized with 3 internal knots at the 10th, 75th, and 90th percentiles of the temperature distributions; ns(Prcep) is daily cumulative precipitation with a natural cubic spline of 3 *df*^21^; ns(Time) is a term for adjustment of long-term trend and seasonality with natural cubic spline of 7 *df* per year^27,31^; DOW is day of the week; and COVID19 is a binary variable indicating before and after the WHO characterized COVID-19 as a pandemic on March 11, 2020. Reporting_indicator is the reporting system indicator term to represent the change in the reporting system on May 20, 2019. In brief, after the implementation of the change in reporting system, there were several symptoms that ceased to be reported or were reported significantly less often before or after this period. However, this was not the case for gastroenteritis reports because the number of cases did not change (eFigure 3 in Supplement 1). Nevertheless, this term was included to account for potential unmeasured effects due to reporting. An adjustment was made for the log of the population to account for potential population changes, along with incorporating the previous day’s number of gastroenteritis cases, which is believed to be strongly correlated with same-day cases. Residuals of the parameterized model showed indications of homoscedasticity (eFigure 4 in Supplement 1). In this study, low and high temperature effects, relative to the minimum risk temperature (MRT), are represented by the 10th and 90th temperature percentiles, respectively. The concept of MRT draws inspiration from temperature-mortality studies’ minimum mortality temperature. As the name implies, MRT represents the minimum temperature at which the risk, whether associated with mortality or morbidity, is at its lowest. Furthermore, lagged association was examined in both the 10th and 90th temperature percentiles of the respective camps (eFigure 5 in Supplement 1).

All statistical analyses were conducted using R statistical software, version 4.3.2, with the packages dlnm and glm (R Project for Statistical Computing). Statistical significance was defined with *P* < .05.

For a sensitivity analysis, temperature, precipitation, and long-term trend degrees of freedom, as well as the maximum number of lag days, were varied in assessing the association with the risk curve. Maximum lags were also varied between 7 and 35 days to understand how the risk curve changes and assess the optimal maximum lag subject to a viable interpretation of the results.^32^ The quasi-Akaike information criterion (qAIC) was calculated and used to assess each generated quasi-Poisson generalized linear model for goodness of fit.^33^

## Results

### Descriptive Statistics

**Figure 1.**
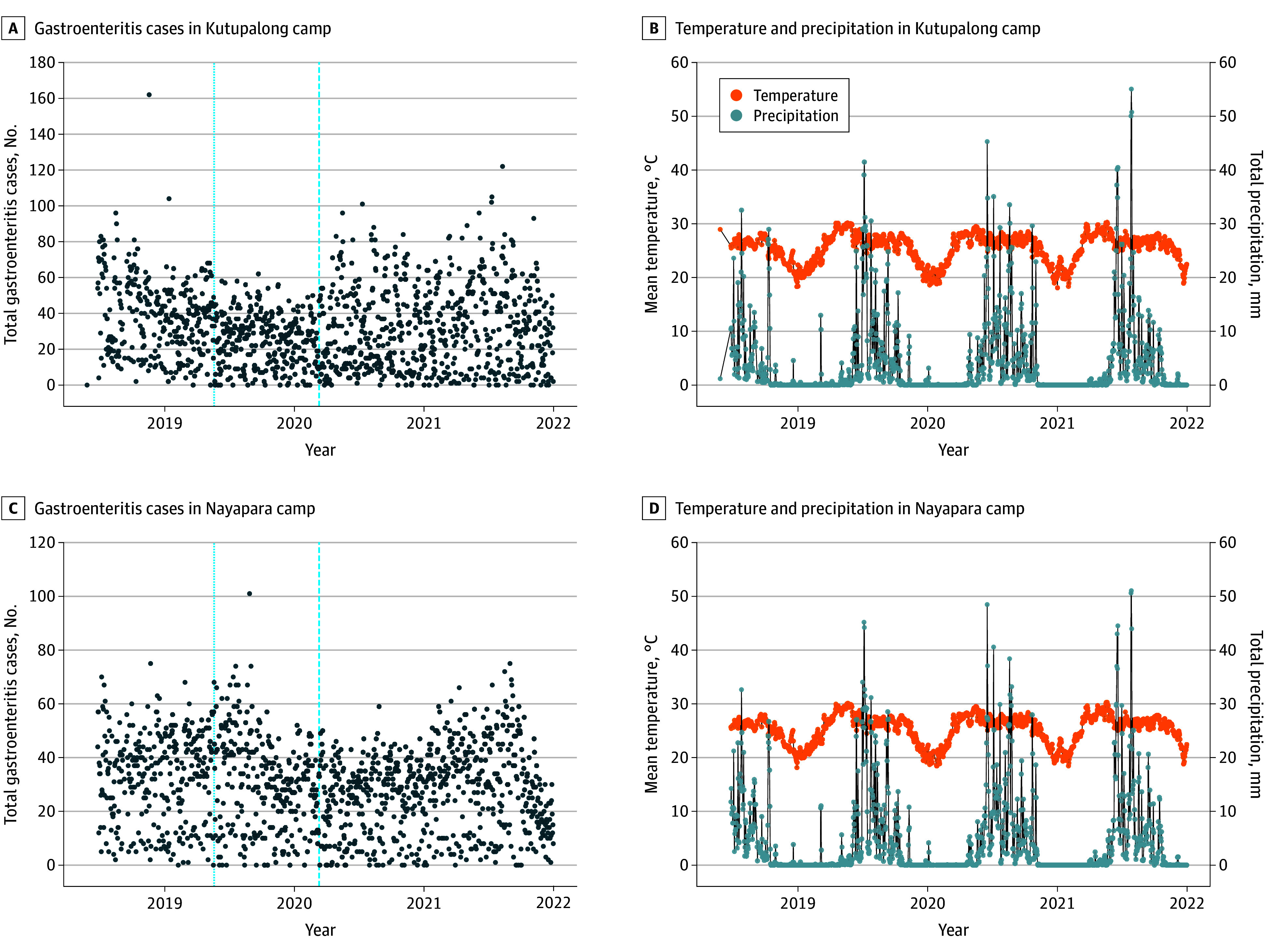
Time Series of the Number of Gastroenteritis Cases, Daily Mean Temperature, Daily Precipitation, 2019-2021 A, Gastroenteritis cases in Kutupalong camp. B, Temperature and precipitation in Kutupalong camp. C, Gastroenteritis cases in Nayapara camp. D, Temperature and precipitation in Nayapara camp. Reporting indicator on May 20, 2019, with blue dotted line; COVID-19 pandemic declaration by World Health Organization on March 11, 2020, with blue dashed line.

A total of 33 280 gastroenteritis cases (95% among individuals aged ≥5 years; 71% female and 29% male) were recorded in Kutupalong and 31 165 gastroenteritis cases (99% among individuals aged ≥5 years; 67% female and 33% male) were recorded in Nayapara (Table). A significant majority, 95%, of gastroenteritis cases were observed in individuals aged 5 years or older, with the remaining 5% occurring among those aged 4 years or younger. Temperature and precipitation patterns (Figure 1) were identical for both camps, with temperatures of approximately 26 °C. Seasonality was apparent for both temperature and total precipitation with nearly similar peaks, with temperature peaking around May just 2 months before the peak of precipitation in July.

**Table.** Descriptive Summary of the Gastroenteritis Cases in Kutupalong and Nayapara Camps

Characteristic	Kutupalong UNHCR-RHU clinic			Nayapara UNHCR-RHU clinic		
	No. of cases (%)	No. of cases, daily mean (SD)	No. of cases, median (IQR)	No. of cases (%)	No. of cases, daily mean (SD)	No. of cases, median (IQR)
All cases	33 280 (100)	30.5 (20.8)	29 (13-43.25)	31 165 (100)	28.8 (16.3)	30 (15-40)
Age group, y^a^						
≤4	1650 (5)	1.5 (2.7)	0 (0-2)	224 (1)	0.2 (1.0)	0 (0-0)
≥5	31 582 (95)	28.9 (20.1)	28 (12-41)	30 888 (99)	28.5 (16.2)	30 (15-40)
Sex						
Male	9525 (29)	8.7 (7.3)	7 (3-12)	10 399 (33)	9.6 (6.1)	9 (5-14)
Female	23 755 (71)	21.7 (15.3)	21 (8-31)	20 766 (67)	19.2 (10.9)	20 (10-27)

Abbreviation: UNHCR-RHU, Refugee Housing Unit of United Nations High Commissioner for Refugees.

^a^ There are records where age data were not collected, including 48 cases in the Kutupalong clinic and 53 cases in the Nayapara clinic with unknown age.

### Exposure-Response Association

The 2 camp clinics exhibited varying cumulative temperature-gastroenteritis risk patterns. In Kutupalong, the risk curve resembled a U-shaped pattern, whereas in Nayapara, the risk of gastroenteritis increased as the temperature decreased. To facilitate a comparison between the temperature-gastroenteritis risk curves of both camps, MRT was set at 26 °C.

**Figure 2.**
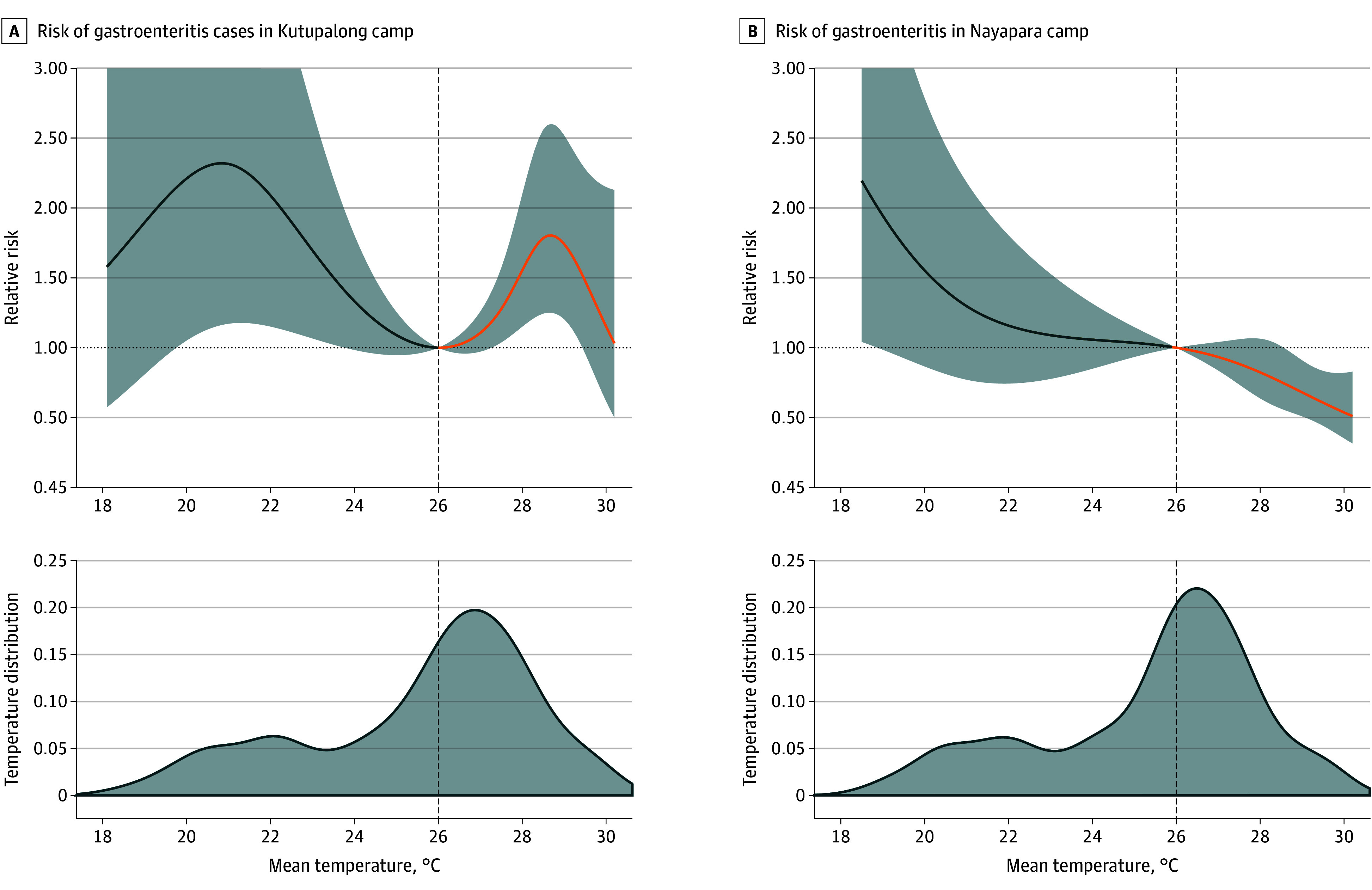
Temperature and Gastroenteritis Risk Curves in Kutupalong and Nayapara Camp Sites, Cox’s Bazar, Bangladesh Overall cumulative summary and the 95% CI are reported as shaded areas. The dashed vertical lines indicate the minimum risk temperature at 26 °C in both Kutupalong (A) and Nayapara (B). The blue line indicates lower temperature risks, whereas the orange line indicates higher temperature risks, relative to the minimum-risk temperature. The bottom shaded areas indicate the temperature distribution in Kutupalong camp (A) and Nayapara camp (B).

The temperature-gastroenteritis association and the temperature distribution are shown in Figure 2. In Kutupalong, the relative risk (RR) at the 10th temperature percentile (21.1 °C) was 2.31 (95% CI, 1.18-4.65), whereas the RR at the 90th temperature percentile (28.5 °C) was 1.78 (95% CI, 1.24-2.56), both relative to the MRT. In Nayapara, increased risk of gastroenteritis was associated with with increasing temperature. The RR at the 10th temperature percentile (21 °C) was 1.32 (95% CI, 0.78-2.24), while the RR at the 90th temperature percentile (28.3 °C) was 0.75 (95% CI, 0.56-0.99), relative to the MRT.

Amid varying degrees of freedom and maximum lags, the risk curves remained minimally affected. In particular, there were no substantial differences in the qAIC between the models; qAICs are described in eTables 1 to 4 in Supplement 1.

### Lag-Response Association

**Figure 3.**
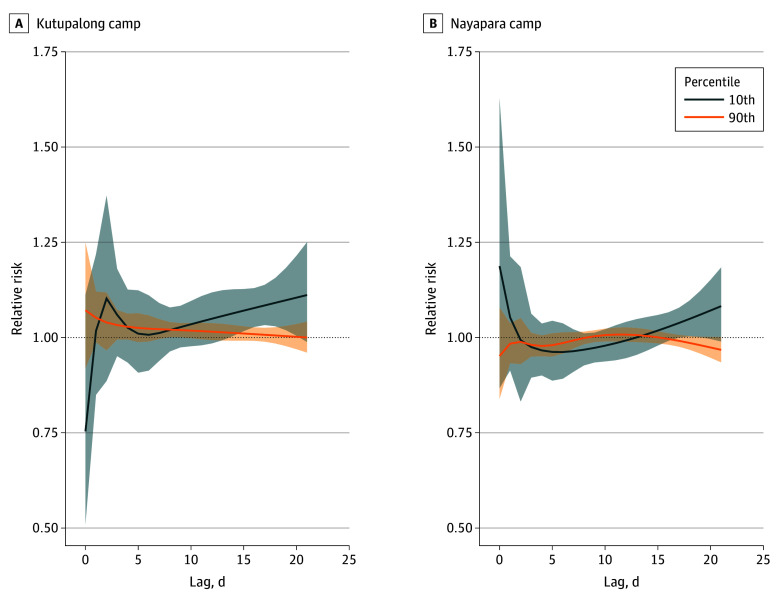
Lag-Response Curves for Forcibly Displaced Persons in Kutupalong and Nayapara at the 10th and 90th Temperature Percentiles Relative to the Minimum Risk Temperature The blue and orange lines indicate the central estimates, and the shaded areas indicate the 95% CIs. The horizontal dotted line at a relative risk of 1.00 indicates the null association. The lag-response association is examined per day with a maximum length of 21 days.

In Kutupalong, cold temperature (10th percentile), although noticeable at around 2 days of onset, was associated with a statistically significant risk of gastroenteritis at around 15 to 20 days (range; RR, 1.06 [95% CI, 1.00-1.13] to RR, 1.10 [95% CI, 1.00-1.21]), as shown in Figure 3. On the other hand, a borderline and marginal increment in risk was associated with warm temperatures (90th percentile) at a lag of 8 days (RR, 1.02 [95% CI, 1.00-1.04]). In Nayapara (Figure 3), a similar association was observed between cold temperatures and an immediately increased risk of gastroenteritis, although statistically not significant, for same-day exposure. Gastroenteritis risks were correspondingly higher at longer lags (lag, 18 days; RR, 1.05 [95% CI, 1.00-1.10]). Warm temperatures were not associated with risk of gastroenteritis across the lag range, with the lag-response curve hovering around the null.

## Discussion

To our knowledge, this study is the first attempt to evaluate the association of temperature with gastroenteritis in the context of FDPs in refugee camps. Here, temperature-associated gastroenteritis risk varied across the 2 large Rohingya refugee camps in Bangladesh. In both Kutupalong and Nayapara, gastroenteritis risks were observed to increase as temperatures decreased. However, beyond the MRT, there was no consistent pattern of temperature-associated gastroenteritis risk.

Our findings suggest a heightened gastroenteritis risk associated with both low and high temperatures, with noted inconsistencies. Studies conducted in Asia,^21,29,34,35,36^ Africa,^37,38,39^ South America,^40,41^ and Oceania^42^ have shown that higher temperatures were associated with elevated gastroenteritis risk. In Bangladesh,^21,36^ increasing temperatures were associated with risk of noncholera gastroenteritis. However, studies in Japan,^32^ China,^43,44,45^ Spain,^46^ and the Philippines^28^ reported increased risk with both low and high temperatures.

Kutupalong exhibited a nonlinear U-shaped risk function, whereas Nayapara’s risk curve was linear (inverse), as shown in Figure 2; both findings are in concurrence with previous studies. In a study in Bangladesh, gastroenteritis risk increased with increasing temperature.^21^ On the other hand, in Japan,^32^ the Philippines,^28^ and Spain,^46^ a U-shaped risk function was found, with increasing gastroenteritis risks with decreasing and increasing temperatures relative to the MRT. Also, an M-shape association with 2 peaks was documented using data from 270 Chinese cities.^45^ The variation in the temperature-gastroenteritis risk function can be partially explained by the role of temperature in the pathogen-specific diarrheal types.^14^ Increased risk of viral gastroenteritis is more prominent at low temperatures, whereas risk of bacterial gastroenteritis is discernible at high temperatures, which has also been reported in a similar pathogen-specific study in Korea.^15^

Consistent in both camps, cold temperatures tended to have a longer lagged association, with gastroenteritis risks peaking at around 18 days (or 2.5 weeks), as shown in Figure 3. Sung and colleagues^15^ documented that at cold temperatures, the risk for viral gastroenteritis was particularly high at around 2 to 3 weeks from onset. Similarly, the lagged effect for cold temperatures may be mostly associated with the specific pathogen that causes gastroenteritis. In this case, viral pathogens tend to thrive better in colder conditions where prognosis can be longer at around 2 weeks. Warmer temperatures deter viral pathogens; however, they can be conducive for bacterial pathogens to be more active, with a typical prognosis lasting from a couple of days to a week. A similar phenomenon was noted in Kutupalong, where warm temperature–related gastroenteritis risks peaked at a lag of 8 days. In Spain, Morral-Puigmal and colleagues^46^ noted an even shorter period at around 1 to 5 days, with the warm temperature–related risk of gastroenteritis hospitalizations peaking on the same day and thereafter decreasing until the risk approached the null on the sixth day. The authors furthermore found that foodborne gastroenteritis, typically bacterial in nature, was associated with warm temperatures.

The results of these epidemiologic studies are consistent with the associations reported in in vitro studies. For bacterial pathogens such as *Salmonella*, *Shigella*, *Campylobacter*, and *E coli*, several in vitro research studies found that these bacteria grow optimally at around 37 °C, and all of these bacteria can survive at temperatures higher than 40 °C.^47,48,49,50,51^ Factors other than temperature are also associated with bacterial survival. Although isolates have shown reduced survival at high temperatures,^52,53,54,55^ increases in these bacteria at high temperatures have been reported in natural environments, such as in food. Unlike bacterial pathogens, rotavirus and norovirus generally survive better at low rather than high temperatures.^56,57^ For norovirus, viral infectivity was significantly reduced at room temperature compared with at 4 °C.^58,59^

### Strengths and Limitations

This study has some strengths. It is the first study that examined the association between gastroenteritis and temperature among FDPs. Although health challenges arising from vulnerability and exposure to climatic conditions in FDP camps are becoming increasingly concerning,^60,61^ reports examining the association between environmental factors and the health conditions at camp sites are still relatively scarce. Although there is 1 study in Ethiopia that examined the cross-sectional association between gastroenteritis and diarrhea among displaced populations,^62^ the time-unadjusted results may prove to have limited interpretation due to the cross-sectional nature of the study. In contrast, our study provides, apart from an increased statistical power due to an increased number of observations, a more robust examination of the associations of temperature with gastroenteritis after adjusting for temporally changing, be it short-term or long-term, trends that may confound the association.

This study also has some limitations. Although our study has provided an in-depth examination of the association between temperature and risk of gastroenteritis in FDP camps, the results of this study are limited to the context of Rohingya camps. Living conditions, such as the presence or absence of a sanitary environment, as well as the existing vulnerability of the population, may vary between camps, which may modify the association.^60,63^ Similarly, due to the absence of camp-specific data, other potential socioeconomic covariates that could influence the association,^64,65^ particularly water, sanitation, and hygiene factors (such as the type of sanitation facility, accessibility to clean water resources, and water treatment practices), were not adjusted in the analysis. Individual-level data, such as comorbidities, were also lacking. These variabilities, spatial, time varying, and time invariant, when included in the analyses, would provide a better understanding of the temperature-gastroenteritis association.

Also, because the health outcome data in this study are derived from symptom-based registries, examination and comparison with other pathogen-specific studies are limited. Likewise, the use of predetermined grouping, such as age, made it not viable to do age-specific analysis. A previous study among Rohingya refugees by Hossain and colleagues^66^ revealed that bacterial and viral pathogens of gastroenteritis varied across age groups. This finding suggests that age-specific characteristics or behaviors may be associated with the risk of gastroenteritis and its potential temperature-related effects; thus, future studies would benefit if anonymized data could be obtained per visit. Furthermore, because this is a vulnerable population–specific study, comparison of the temperature-gastroenteritis risk with that of the host and general populations is a noteworthy topic that remains to be explored at a later time subject to data availability.

Contrary to expectations, there were fewer cases of gastroenteritis in the population younger than 5 years than in the population 5 years or older, which may be due to the dispersed access of the population younger than 5 years to other health facilities apart from those in the study. There are other health facilities that are operating concurrently with the UNCHR clinics that are mostly donor operated. There is potential that the cases that were reported in the other clinics had more patients younger than 5 years than the UNCHR clinics. However, due to the nature of these clinics, with most of them having ceased to exist due to discontinuation of funding during the COVID-19 pandemic, we were not able to fully examine the effect of this selection bias on the current association.

Last, the data of 2 clinics in Cox’s Bazar were used. Health data were collected across multiple clinics by multiple camps and organizations. However, there were only 2 datasets collected from 2 clinics organized by the government of Bangladesh continuously over 3 years, due to the loss of temporary clinics and restrictions on internal and external movement imposed by the COVID-19 pandemic.^67^ Increasing the number of camp sites, subject to data availability, would be ideal in further providing more robust risk function shapes.

## Conclusions

In this cross-sectional study, air temperature was found to be associated with gastroenteritis, with risks noted to be higher at lower temperatures among FDPs. As the number of FDPs will prospectively increase in the future, determining the extent of how weather-related factors are associated with their health conditions is an important milestone in climate proofing the forcibly displaced camp’s health response.

## Notes

Supplement 1. **eFigure 1.** Map of Cox’s Bazar (Right: Enlargement) in Bangladesh (Left), the Study Area **eFigure 2.** Correlation of Daily Mean Temperatures Between NOAA Monitoring Data and ERA5-Land Reanalysis Data at Shah Amanat International Airport, Using Date and Temperature With Generalized Linear Models for NOAA and ERA5-Land Each Daily Mean Temperature Data **eFigure 3.** Bar Plots for Each Covariate **eFigure 4.** Residual Plots in Generarized Linear Model With DLNM to Analyze Association Between Temperature and Gastroenteritis **eFigure 5.** Temperature-Gastroenteritis Association Displayed the Bidimensional Surface of the Fitted Relative Risk (RR) in a 3-D Graph, Predicted for the Grid of Temperature and Lag Values **eTable 1.** The qAIC for the Number of Knots in Cross-Basis Function of Temperature in DLNM **eTable 2.** The qAIC for the Maximum Lag Days in Cross-Basis Function of Temperature in DLNM **eTable 3.** The qAIC for the Degree of Freedom in Natural-Cubic Spline of Precipitation **eTable 4.** The qAIC for the Degree of Freedom/Year in Natural-Cubic Spline of Seasonality
eReferences.

Supplement 2.
Data Sharing Statement
