# Supplementary material for: Air Temperature and Gastroenteritis Among Rohingya Populations in Bangladesh Refugee Camps
Source: JAMA Netw Open. 2025 Apr 18;8(4):e255768. doi: 10.1001/jamanetworkopen.2025.5768 (PMC12008762; doi:10.1001/jamanetworkopen.2025.5768)
Supplement: Supplement 1. — eFigure 1. Map of Cox’s Bazar (Right: Enlargement) in Bangladesh (Left), the Study Area eFigure 2. Correlation of Daily Mean Temperatures Between NOAA Monitoring Data and ERA5-Land Reanalysis Data at Shah Amanat International Airport, Using Date and Temperature With Generalized Linear Models for NOAA and ERA5-Land Each Daily Mean Temperature Data eFigure 3. Bar Plots for Each Covariate eFigure 4. Residual Plots in Generarized Linear Model With DLNM to Analyze Association Between Temperature and Gastroenteritis eFigure 5. Temperature-Gastroenteritis Association Displayed the Bidimensional Surface of the Fitted Relative Risk (RR) in a 3-D Graph, Predicted for the Grid of Temperature and Lag Values eTable 1. The qAIC for the Number of Knots in Cross-Basis Function of Temperature in DLNM eTable 2. The qAIC for the Maximum Lag Days in Cross-Basis Function of Temperature in DLNM eTable 3. The qAIC for the Degree of Freedom in Natural-Cubic Spline of Precipitation eTable 4. The qAIC for the Degree of Freedom/Year in Natural-Cubic Spline of Seasonality eReferences. [file jamanetwopen-e255768-s001.pdf]

Supplementary Online Content

Takata T, Seposo X, Hossain N, Ueda K. Air temperature and gastroenteritis among Rohingya populations in Bangladesh refugee camps. *JAMA Netw Open.* 2025;8(4):e255768. doi:10.1001/jamanetworkopen.2025.5768

eFigure 1. Map of Cox's Bazar (Right: Enlargement) in Bangladesh (Left), the Study Area
eFigure 2. Correlation of Daily Mean Temperatures Between NOAA Monitoring Data and ERA5-Land Reanalysis Data at Shah Amanat International Airport, Using Date and Temperature With Generalized Linear Models for NOAA and ERA5-Land Each Daily Mean Temperature Data
eFigure 3. Bar Plots for Each Covariate
eFigure 4. Residual Plots in Generarized Linear Model With DLNM to Analyze Association Between Temperature and Gastroenteritis
eFigure 5. Temperature-Gastroenteritis Association Displayed the Bidimensional Surface of the Fitted Relative Risk (RR) in a 3-D Graph, Predicted for the Grid of Temperature and Lag Values
eTable 1. The qAIC for the Number of Knots in Cross-Basis Function of Temperature in DLNM
eTable 2. The qAIC for the Maximum Lag Days in Cross-Basis Function of Temperature in DLNM
eTable 3. The qAIC for the Degree of Freedom in Natural-Cubic Spline of Precipitation
eTable 4. The qAIC for the Degree of Freedom/Year in Natural-Cubic Spline of Seasonality
eReferences.

This supplementary material has been provided by the authors to give readers additional information about their work.

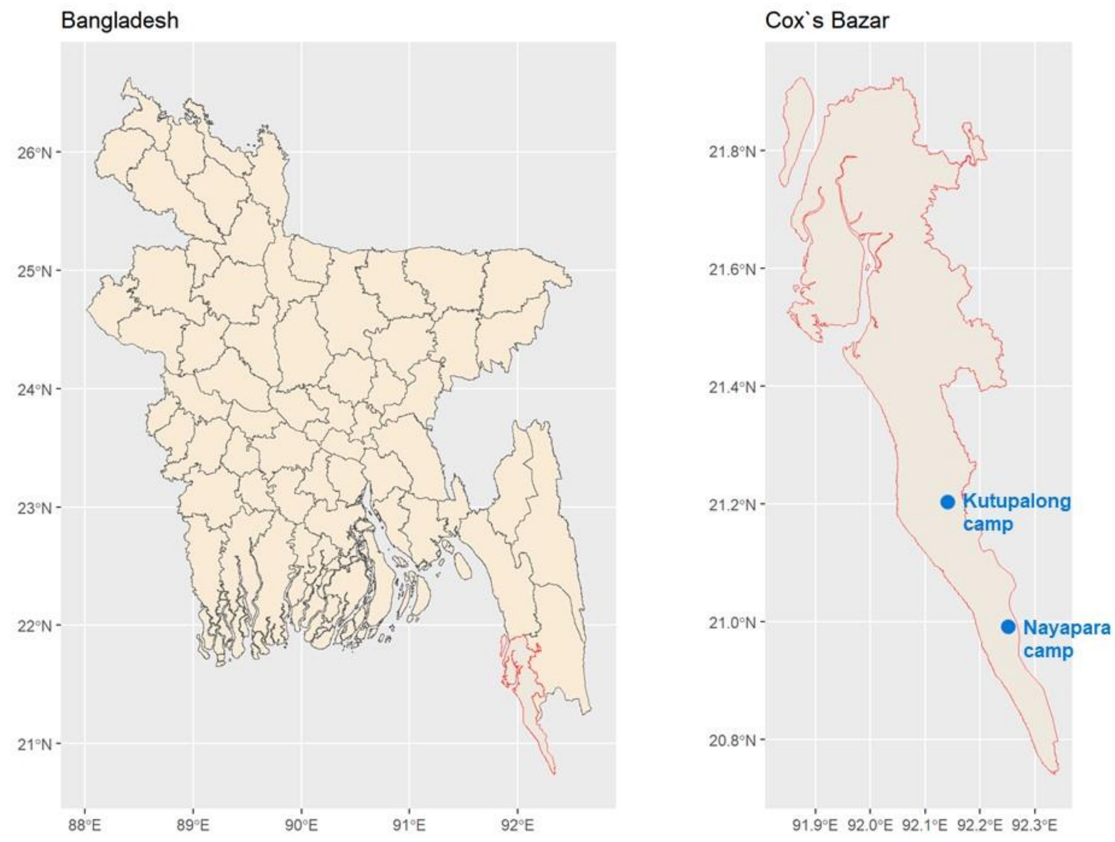

eFigure 1. Map of Cox's Bazar (Right: Enlargement) in Bangladesh (Left), the Study Area. The camp where the clinic, data were collected, is located in Cox's Bazar (right) is marked with a blue dot; Kutupalong clinic (North 21.20°, East 92.15°) and for the Nayapara clinic (North 20.95°, East 92.25°).

© 2025 Takata T et al. *JAMA Network Open.*

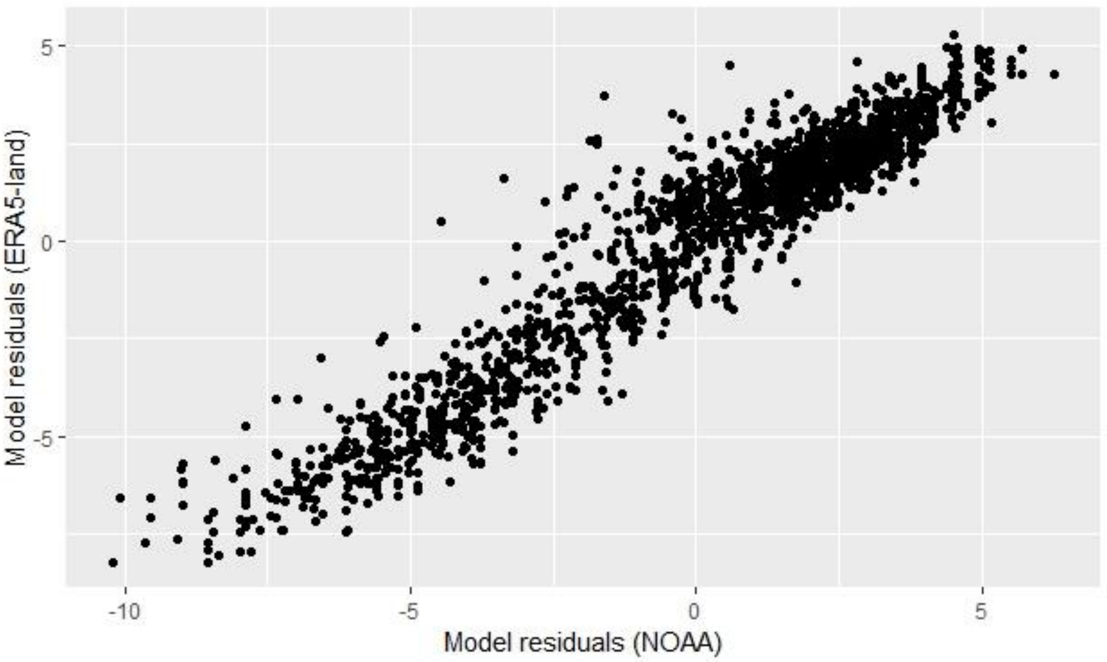

eFigure 2. Correlation of Daily Mean Temperatures Between NOAA Monitoring Data and ERA5-Land Reanalysis Data at Shah Amanat International Airport, Using Date and Temperature With Generalized Linear Models for NOAA and ERA5-Land Each Daily Mean Temperature Data. Correlations were tested using model residues to adjust for correlations due to seasonality. The Pearson's correlation coefficient was approximately $r = 0.955$. The monitoring station at shar amanat international airport was the closest station to the study campsite with few missing data; data from Jan01/2017~May01/2022 were used.

© 2025 Takata T et al. *JAMA Network Open.*

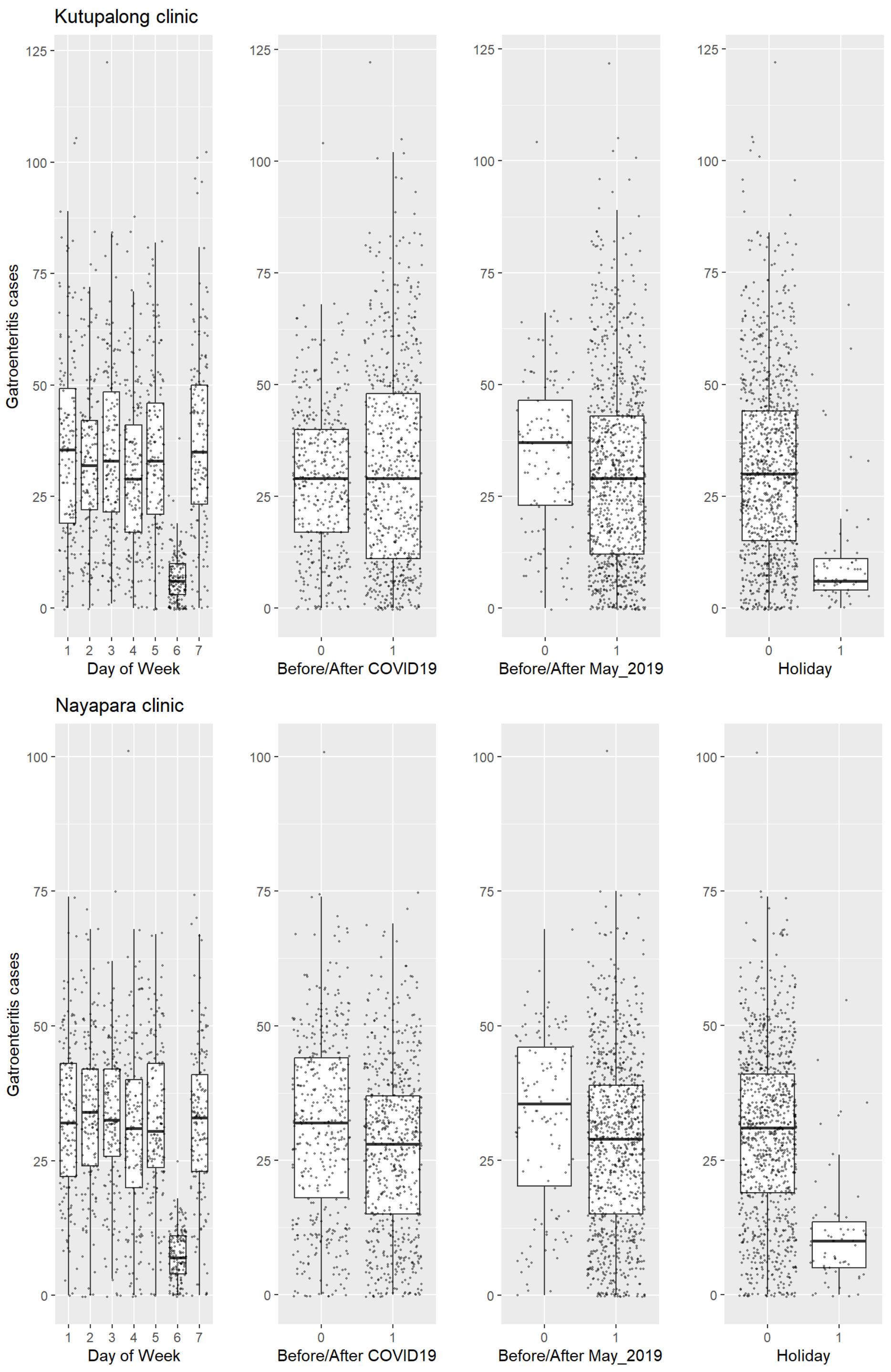

eFigure 3. Bar Plots for Each Covariate. Upper figure: as Kutupalong camp, lower figure: as Nayapara camp. The number of gastroenteritis recipients is shown in relation to each of the factors regarding the day of the week (1~7; Sunday to Saturday), holidays, before and after COVID-19 and the Reporting_indicator (May 2019), which were set as covariates. Friday, the sixth day of the week, is the weekend in Bangladesh.

© 2025 Takata T et al. *JAMA Network Open.*

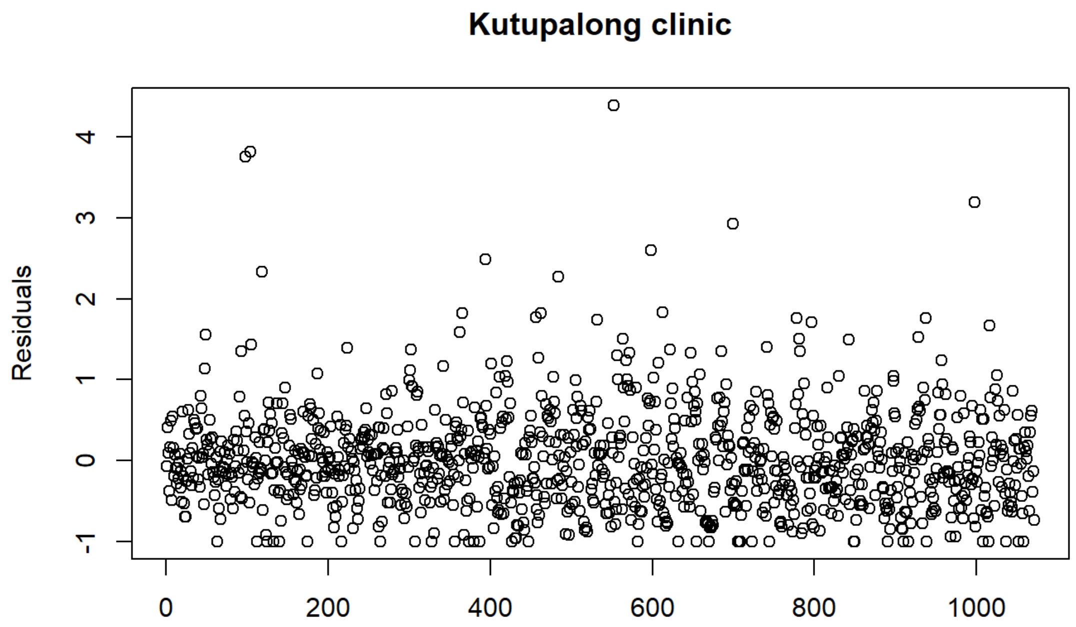

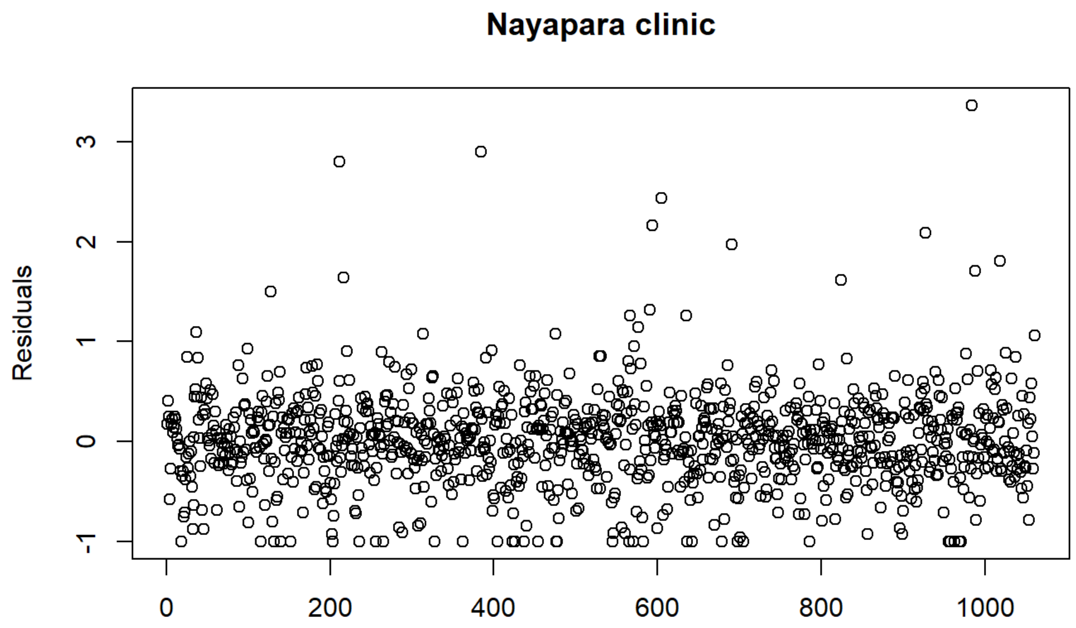

eFigure 4. Residual Plots in Generarized Linear Model With DLNM to Analyze Association Between Temperature and Gastroenteritis. Upper figure: as Kutupalong camp, lower figure: as Nayapara camp.

© 2025 Takata T et al. *JAMA Network Open.*

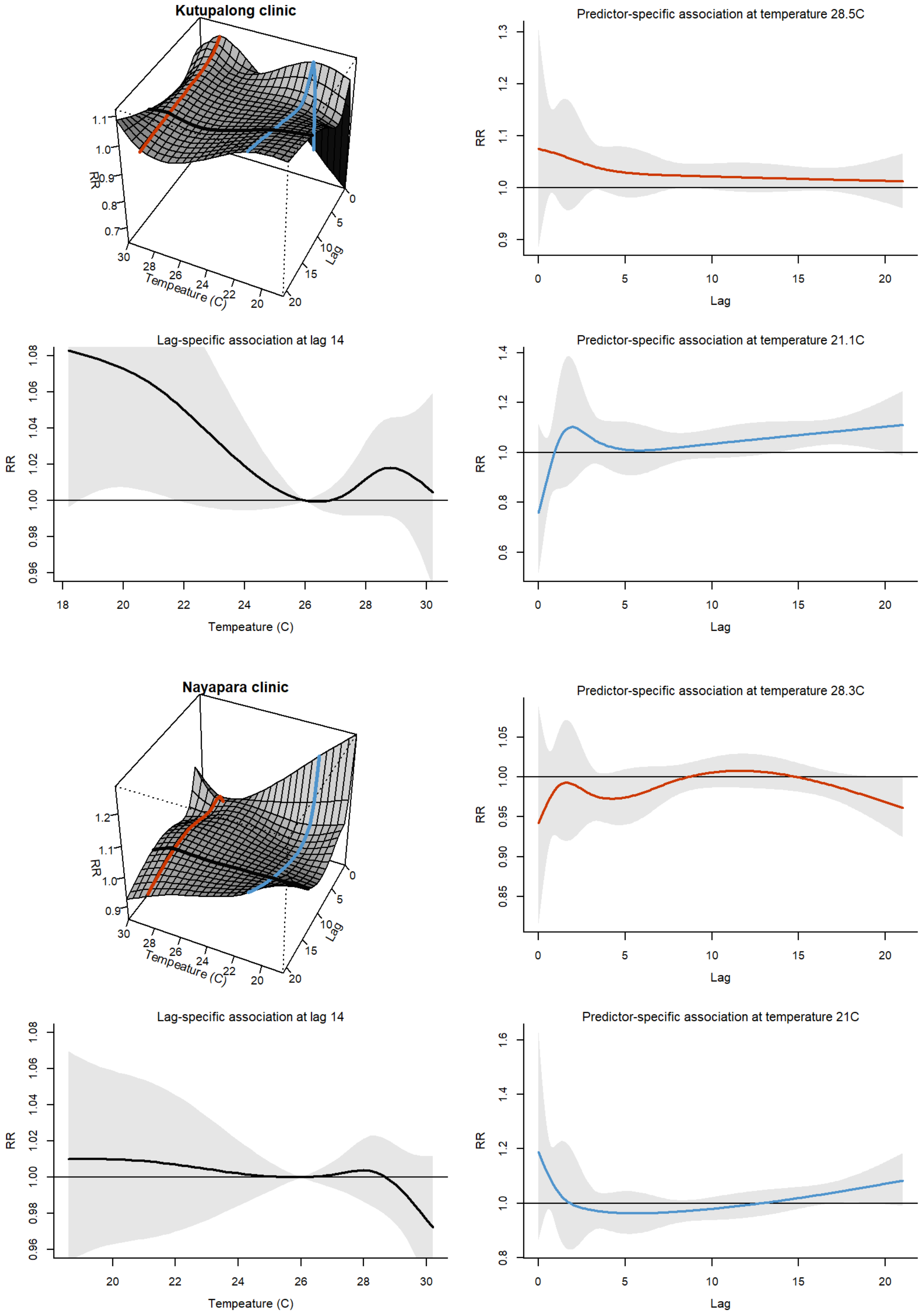

eFigure 5. Temperature-Gastroenteritis Association Displayed the Bidimensional Surface of the Fitted Relative Risk (RR) in a 3-D Graph, Predicted for the Grid of Temperature and Lag Values. Upper

© 2025 Takata T et al. *JAMA Network Open.*

figure: as Kutupalong camp, lower figure: as Nayapara camp. Top-left: 3-D graph. Top-right: predictor-specific summary at 90th percentile (red line parallel to the reference in the 3-D graph). Bottom-left: lag-specific summary at lag 14 (black line perpendicular to the reference in the 3-D graph). Bottom-right: predictor-specific summary at 10th percentile (blue line parallel to the reference in the 3-D graph) The 95%CI are reported as grey areas.

© 2025 Takata T et al. *JAMA Network Open.*

The qAIC for degree of freedom and lag days in generarized linear model with DLNM. The qAIC (quasi-AIC) is used to evaluate quasi-Poisson models.[1] In this study, quasi-Poisson models with DLNM are used and qAIC is calculated for sensitivity analysis. The values covered in grey in each table are the settings of the models used within this study.

eTable 1. The qAIC for the Number of Knots in Cross-Basis Function of Temperature in DLNM. The number of internal knots in the model of this study used is 3knots. In previous studies, 2knots to 3knots have been used. As the association between high and low temperatures is known,[2] a setting of 2knots[3] or 3knots[4] has been used in response to them.

Model of Kutupalong		Model of Nayapara	
The number of knots in cross-basis function about temperature	qAIC	The number of knots in cross-basis function about temperature	qAIC
3 knots	15476	3 knots	11163
2 knots	15546	2 knots	11140

© 2025 Takata T et al. *JAMA Network Open.*

eTable 2. The qAIC for the Maximum Lag Days in Cross-Basis Function of Temperature in DLNM

Model of Kutupalong		Model of Nayapara	
Maximum lag days in cross-basis function (days)	qAIC	Maximum lag days in cross-basis function (days)	qAIC
7	15762	7	11441
14	15623	14	11272
21	15476	21	11163
28	15358	28	11116
35	15032	35	10996

© 2025 Takata T et al. *JAMA Network Open.*

eTable 3. The qAIC for the Degree of Freedom in Natural-Cubic Spline of Precipitation

Model of Kutupalong		Model of Nayapara	
Degree of freedom in natural-cubic spline about precipitation	qAIC	Degree of freedom in natural-cubic spline about precipitation	qAIC
1	15459	1	11149
2	15477	2	11153
3	15476	3	11163
4	15424	4	11163
5	15443	5	11165

© 2025 Takata T et al. *JAMA Network Open.*

eTable 4. The qAIC for the Degree of Freedom/Year in Natural-Cubic Spline of Seasonality

Model of kutupalong		Model of Nayapara	
Degree of freedom/ year of seasonality	qAIC	Degree of freedom/ year of seasonality	qAIC
2	15544	2	11162
3	15544	3	11159
4	15492	4	11137
5	15497	5	11160
6	15440	6	11132
7	15476	7	11163
8	15389	8	11164
9	15426	9	11132

© 2025 Takata T et al. *JAMA Network Open.*

eReferences.

1. Gasparrini A, Armstrong B. Reducing and meta-analysing estimates from distributed lag non-linear models. *BMC medical research methodology*. 2013;13(1):1-10.
2. Carlton EJ, Woster AP, DeWitt P, Goldstein RS, Levy K. A systematic review and meta-analysis of ambient temperature and diarrhoeal diseases. *International journal of epidemiology*. 2016;45(1):117-130.
3. Chua PL, Ng CFS, Madaniyazi L, et al. Projecting Temperature-Attributable Mortality and Hospital Admissions due to Enteric Infections in the Philippines. *Environmental health perspectives*. 2022;130(2):027011.
4. Onozuka D, Gasparrini A, Sera F, Hashizume M, Honda Y. Modeling Future Projections of Temperature-Related Excess Morbidity due to Infectious Gastroenteritis under Climate Change Conditions in Japan. *Environmental Health Perspectives*. Jul 2019;127(7)077006. doi:10.1289/ehp4731

© 2025 Takata T et al. *JAMA Network Open.*
