# Supplementary material for: Air Temperature and Gastroenteritis Among Rohingya Populations in Bangladesh Refugee Camps
Source: JAMA Netw Open. 2025 Apr 18;8(4):e255768. doi: 10.1001/jamanetworkopen.2025.5768 (PMC12008762; doi:10.1001/jamanetworkopen.2025.5768)
Supplement: Supplement 2. — Data Sharing Statement [file jamanetwopen-e255768-s002.pdf]

Data Sharing Statement

Takata. Air Temperature and Gastroenteritis Among Rohingya Populations in Bangladesh Refugee Camps. *JAMA Netw Open*. Published April 18, 2025.
doi:10.1001/jamanetworkopen.2025.5768

Data
Data available: No

Additional Information
Explanation for why data not available: The data can be access through the DHIS2 interface (https://dhis2.org/) via proper channels to those who maintain the database.
